# Honey‐Based Sodium Alginate–Polyvinyl Alcohol Hydrogel Containing Green‐Synthesized Chitosan–Zinc Oxide Nanoparticles for Wound Healing

**DOI:** 10.1155/bca/9989560

**Published:** 2025-12-28

**Authors:** Nahid Shahabadi, Saba Zendehcheshm, Fatemeh Khademi

**Affiliations:** ^1^ Department of Inorganic Chemistry, Faculty of Chemistry, Razi University, Kermanshah, Kermanshah, Iran, razi.ac.ir; ^2^ Medical Biology Research Center, Health Technology Institute, Kermanshah University of Medical Sciences, Kermanshah, Iran, kums.ac.ir

**Keywords:** antibacterial hydrogel, chitosan, green synthesis, honey, wound healing, ZnO NPs

## Abstract

Chronic wounds remain a global health challenge, necessitating advanced materials and methods for effective treatment. Nanotechnology offers promising solutions by enabling innovative wound care strategies. This study presents an antibacterial hydrogel composed of polyvinyl alcohol (PVA)/sodium alginate (SA)/honey (PSH) embedded with chitosan (CH)‐coated zinc oxide nanoparticles (ZnO NPs‐CH). The ZnO NPs were synthesized via a green method using phytochemicals from *Cerasus microcarpa* (wild cherry) wood extract, then coated with CH to enhance antibacterial properties. These NPs were incorporated into the PSH matrix to form a novel nanocomposite hydrogel (PSH/ZnO NPs‐CH). Characterization using FE‐SEM, EDX, and ATR‐FTIR confirmed successful integration and revealed a porous structure beneficial for water absorption and gas exchange. Swelling tests indicated controlled absorption in the ZnO NPs‐CH hydrogel, suitable for exudative wounds. Antibacterial assays showed strong activity against *Escherichia coli* (*E. coli*) and *Staphylococcus aureus* (*S. aureus*). *In vivo* wound healing studies demonstrated enhanced tissue regeneration and reduced inflammation. Overall, the PSH/ZnO NPs‐CH hydrogel shows promise as a multifunctional wound dressing. However, further evaluations are necessary to confirm the reliability and validity of these findings.

## 1. Introduction

Chronic infected wounds pose significant health challenges and result in substantial financial burdens for both patients and healthcare systems. These wounds experience slower healing compared to typical wounds due to the presence of bacterial colonies that form polymicrobial biofilms. Various treatments have been investigated for such infections, including antibiotics, heavy metal compounds, ammonium derivatives, and antimicrobial peptides. However, the effectiveness of antibiotics is increasingly undermined by the rise of resistant bacterial strains, and their use can occasionally cause toxicity in healthy tissues. When antibiotics fail, bacteria can establish and proliferate within biofilms, further complicating treatment. Alternative strategies such as heavy metals and natural extracts have also been explored, but their clinical utility is limited due to toxicity to normal cells. Antimicrobial peptides show promise, yet their high production costs and complex manufacturing processes restrict widespread use. Moreover, quaternary ammonium compounds may lead to drug resistance after prolonged application, similar to antibiotics. Therefore, there is an urgent need to develop innovative strategies that can effectively eradicate bacteria, promote wound healing, and minimize health risks such as side effects and toxicity [1]. In this context, nanotechnology, particularly the application of metallic nanoparticles (NPs), has emerged as a promising solution.

Among metallic NPs, zinc oxide nanoparticles (ZnO NPs) are particularly attractive due to their biocompatibility and broad‐spectrum antibacterial activity. However, conventional chemical synthesis methods often rely on hazardous reagents and raise environmental concerns. Phyto‐nanotechnology, which employs plant extracts as natural reducing and stabilizing agents, offers a safer and cost‐effective alternative while simultaneously enhancing the biological activity of the resulting NPs [2]. ZnO NPs have been increasingly used in wound healing applications owing to their antibacterial and tissue regeneration properties [3–5]. Recent studies suggest that the combination of ZnO NPs with chitosan (CH) can further enhance antibacterial performance. This synergistic effect increases the positive charge on CH’s amine groups, improving their ability to bind anionic components on bacterial cell surfaces. Due to its safety, biodegradability, and biocompatibility, CH is frequently used as an antibacterial coating. Additionally, CH accelerates the wound healing process by promoting re‐epithelialization and skin regeneration [6].

In the search for sustainable synthesis routes, medicinal plants play a crucial role. *Cerasus microcarpa* (wild cherry), locally known as “Baraloi” or “Baraluk,” is a native species of western Iran with a long history of traditional medicinal use [7]. In this study, for the first time, the wood extract of this plant was employed for the green synthesis of ZnO NPs.

Beyond NPs, hydrogels represent another important class of materials for wound care. Hydrogels are three‐dimensional (3D) polymeric networks [8] formed through hydrogen bonding or covalent crosslinking, and they have proven highly effective as wound dressings. Their ability to absorb large amounts of liquid creates a moist environment that supports cell proliferation and accelerates wound healing, while their biocompatibility, biodegradability, and adhesiveness contribute to effective tissue repair [9].

Furthermore, the hydrophilic groups within hydrogel polymer chains enable high water retention, which enhances porosity, softness, and elasticity. This hydration also provides a cooling effect, reducing pain during dressing removal. Hydrogels may be derived from natural polymers such as gelatin, starch, CH, cellulose, hyaluronic acid, and SA, or from synthetic polymers such as polyethylene glycol (PEG), PVA, and polyacrylamide. Hybrid systems that combine natural and synthetic polymers can further improve biological, physicochemical, and mechanical properties, thereby supporting enhanced wound healing [10]. SA, a natural polysaccharide from brown algae, is widely used due to its biocompatibility and biodegradability. Its structure, rich in carboxyl groups, enables controlled drug release and supports wound closure [11]. PVA, a nontoxic and water‐soluble synthetic polymer, is often blended with SA to form stable, biocompatible hydrogel matrices for wound healing [12, 13].

In addition to polymeric components, natural bioactive agents such as honey further improve wound healing performance. Honey is a supersaturated sugar solution with antibacterial, antioxidant, and anti‐inflammatory properties. It maintains a moist wound environment, promotes epithelialization, inhibits proteases, releases hydrogen peroxide, and stimulates vascular endothelial growth factor (VEGF) production [10, 14].

Although numerous studies have explored alginate‐ and PVA‐based hydrogels incorporating honey [10, 15, 16], ZnO NPs [17], or silver‐based systems such as Ag/ZnO, lignin‐Ag, and CH‐alginate‐Ag hydrogels [11, 18, 19], the present study introduces three key innovations. For the first time, the wood extract of wild cherry was used for the green synthesis of ZnO NPs, offering an eco‐friendly and sustainable approach for NP fabrication. Second, CH‐coated ZnO NPs were employed to modulate zeta potential, enhance colloidal stability, and improve antibacterial interactions through increased surface cationic charge. Third, unlike some prior studies limited to *in vitro* evaluations, a direct *in vivo* comparison with phenytoin, a clinically approved wound‐healing agent, was conducted, providing a clinically relevant benchmark for assessing the therapeutic efficacy of the PSH/ZnO‐CH hydrogel system. Collectively, these innovations highlight the novelty of our approach and distinguish it from previously reported ZnO‐, Ag‐, and honey‐based hydrogel systems.

In this study, we developed a honey‐based SA/PVA hydrogel incorporating CH‐coated ZnO NPs (PSH/ZnO NPs‐CH hydrogel), synthesized via a plant‐mediated approach using wild cherry wood extract. The NPs and hydrogel were fully characterized, and their antibacterial and wound‐healing performance was directly compared with phenytoin as a clinical reference. This strategy introduces a novel plant‐based synthesis and functional coating approach, providing new insights beyond conventional SA/PVA/ZnO or honey‐loaded hydrogel systems.

## 2. Materials and Methods

### 2.1. Materials

Branches of wild cherry shrub were collected from Shaho Mountain (Kermanshah, Iran) and air‐dried. Zinc nitrate (Zn(NO_3_)_2_·6H_2_O), chitosan (CH, medium molecular weight, viscosity 200–800 cP, 1 wt.% in 1% acetic acid at 25°C, Brookfield; degree of deacetylation 75%–85%; from shrimp shells, Sigma‐Aldrich, Cat. No. 448877, Lot STBC7340V, MDL MFCD00161512), polyvinyl alcohol (PVA, 99%), sodium alginate (SA, M/G = 1.56), calcium chloride (CaCl_2_), glacial acetic acid (≥ 99%), Müller–Hinton agar, and histological reagents (ethanol ≥ 99.9%, 10% formalin, paraffin wax, hematoxylin, and eosin) were purchased from Sigma‐Aldrich. Pure honey was sourced from a beekeeping farm in Ilam, Iran. ZnO NPs were biosynthesized using wood extract of the wild cherry shrub. Gentamicin sulfate discs (10 μg) were obtained from Carl Roth. Bacterial strains *Staphylococcus aureus* (ATCC 25923) and *Escherichia coli* (ATCC 25922) were provided by the Iranian Research Organization for Science and Technology (IROST). Ketamine and xylazine (for anesthesia) were obtained from NexGen Pharmaceuticals.

### 2.2. Preparation of Wild Cherry Shrub Wood Extract

Branches of wild cherry shrub (Figure 1(a)) were collected and air‐dried in the shade to preserve bioactive compounds. Once fully dried, they were crushed into small pieces. Then, 40 g of the crushed material was boiled in 400 mL of double‐distilled water for 1 h under continuous magnetic stirring. As extraction proceeded, the solution gradually turned red, indicating the release of soluble components. After cooling to room temperature, the extract exhibited a pH of approximately 6.2 and was subsequently filtered through Whatman No. 41 filter paper (20–25 μm pore size) to remove residual solids, yielding a clear red filtrate (Figure 1(b)). The filtrate was then transferred into amber glass bottles and stored at 4°C to minimize photodegradation, oxidation of phytochemicals, and microbial growth. Freshly prepared extract was used within 1 week for NP synthesis.

Figure 1(a) Dried branches of wild cherry shrub. (b) Filtration of wild cherry shrub wood extract using a Büchner funnel.

**Figure:**
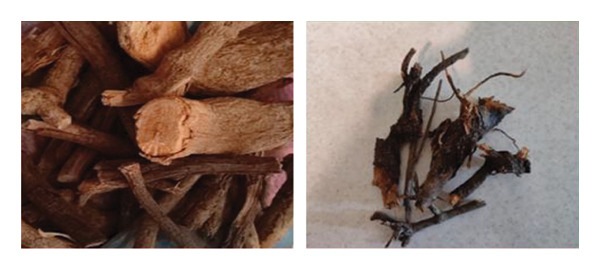
(a)

**Figure:**
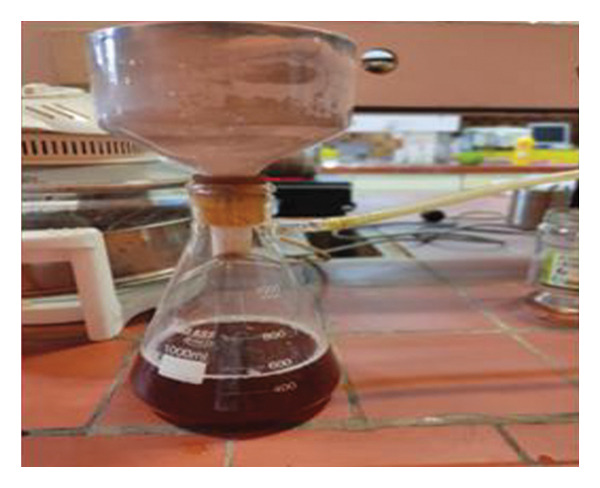
(b)

### 2.3. Biosynthesis of ZnO NPs

ZnO NPs were synthesized using a green route as follows: A 100 mL solution of zinc nitrate Zn(NO_3_)_2_·6H_2_O (0.1 M) was gradually added dropwise into 300 mL of wild cherry shrub wood extract, preheated to 40°C. The mixture was stirred continuously at 400 rpm and heated at a ramp rate of 5°C/min to reach 80°C, where it was maintained for 2 h. During this stage, a gradual color change was observed. The temperature was then increased to 90°C at the same heating rate until the solution volume was reduced to approximately 20 mL, and a viscous gel formed after approximately 1 h. This gel was subsequently transferred to an oven and heated at 100°C overnight, resulting in the formation of a brown precipitate. After drying, the precipitate was calcined at 700°C for 2 h, ultimately yielding fine white ZnO NPs (Figure 2(c), right). The overall yield of ZnO NPs was 75%, calculated as

(1) Yield%=Mass of dried ZnO obtainedTheoretical mass of Zn from zinc nitrate×100.

Figure 2(a) Dispersion of ZnO NPs in double‐distilled water using an ultrasonic device to form a uniform white suspension. (b) The resulting ZnO NPs‐CH mixture after the pH adjustment to 10, forming a stable colloidal suspension with a milky appearance. (c) Digital photograph showing (left) ZnO NPs‐CH powder and (right) ZnO NPs powder.

**Figure:**
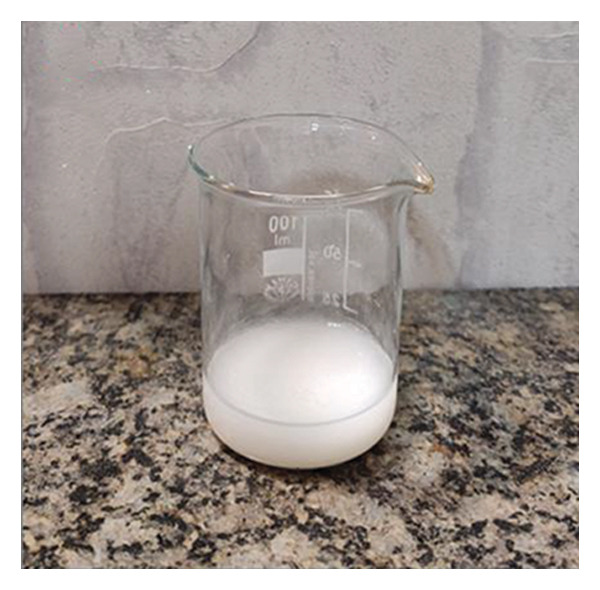
(a)

**Figure:**
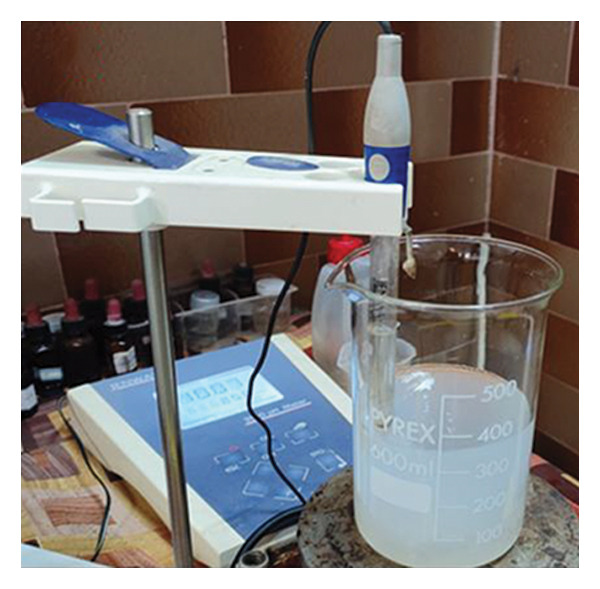
(b)

**Figure:**
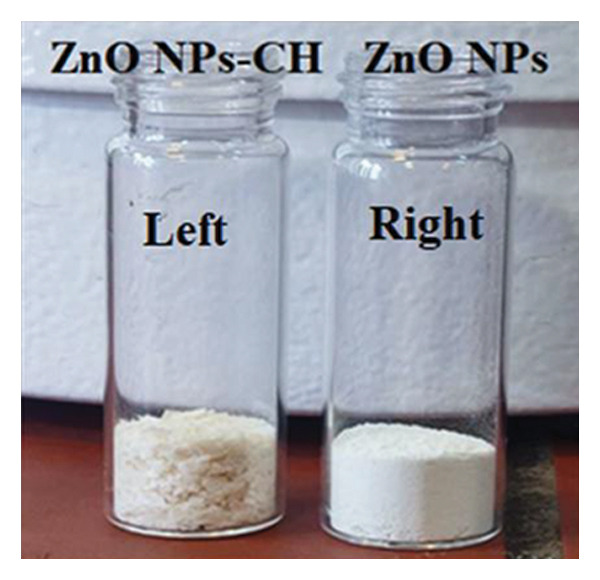
(c)

### 2.4. Preparation of ZnO NPs‐CH

To prepare a 1% acetic acid solution, 2.5 mL of glacial acetic acid was added to 250 mL of double‐distilled water. Then, 0.5 g of CH powder was added and stirred continuously for 24 h to obtain a clear 0.2% CH solution. Next, 0.25 g of green‐synthesized ZnO NPs were dispersed in 20 mL of double‐distilled water using an ultrasonic bath (Elma Elmasonic S 10 H, 90 W, 220–240 V, 50/60 Hz) at full power for 20 min to obtain a uniform white suspension (Figure 2(a)). This suspension was gradually added to the CH solution under continuous stirring. Upon mixing, the color of the solution changed from white to colorless. The pH was adjusted to 10 using a 2 M NaOH solution while monitoring with a pH meter. At this pH, a stable milky colloid formed (Figure 2(b)).

The colloidal suspension was then centrifuged at 10,000 rpm for 15 min at 4°C. The supernatant was carefully discarded, and the precipitate was washed three times with double‐distilled water to remove any unbound CH and residual acetic acid. The collected ZnO NPs‐CH was finally dried in an oven at 60°C for 24 h, yielding a dry powder (Figure 2(c), left).

### 2.5. Characterization of ZnO NPs and ZnO NPs‐CH

In this section, the microscopic and spectroscopic techniques were employed to characterize the synthesized ZnO NPs and ZnO NPs‐CH. Zeta potential measurements were performed using a HORIBA SZ‐100‐Z instrument. Prior to measurement, 10 mg of each sample was sonicated in double‐distilled water for 10 min. UV–vis spectra were recorded using an Agilent 8453 spectrophotometer with a 1 cm path length quartz cuvette, and the background was corrected using the absorption of purified water. The surface morphology of ZnO NPs and ZnO NPs‐CH was analyzed by scanning electron microscopy (SEM, TESCAN BRNO‐Mira3 LMU) operated at an accelerating voltage of 10 kV with a secondary electron detector. For transmission electron microscopy (TEM, Zeiss EM10C, 100 kV), samples were drop‐cast onto carbon‐coated copper grids and air‐dried prior to imaging.

### 2.6. Preparation of PSH/ZnO NPs‐CH Polymeric Hydrogel

**Figure 3:**
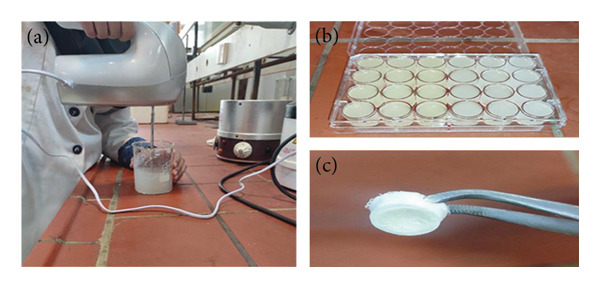
(a) Stirring the SA solution using a high‐speed electric mixer until a uniform and homogeneous gel is obtained. (b) PSH and PSH/ZnO NPs‐CH hydrogels placed in a 24‐well plate before undergoing cross‐linking, freeze–thawing, and freeze cross‐linking processes. (c) The same hydrogel samples after completing these processes, showing uniform initial weights before swelling and CA analysis.

The synthesis of the PVA/SA hydrogel begins with preparing a 10% (w/v) PVA solution by dissolving PVA in double‐distilled water under stirring at 80°C. Simultaneously, a 2% (w/v) SA solution is prepared by dispersing SA in double‐distilled water using a high‐speed stirrer (Figure 3(a)). The two solutions are mixed at a 60 : 40 (v/v) ratio (PVA : SA) and stirred overnight. Then, honey is added to the mixture at a final concentration of 10% (v/v) and stirred at room temperature for 12 h. Meanwhile, ZnO NPs‐CH (2% w/v) are dispersed in double‐distilled water and gradually added to the PSH solution, followed by 2 h of stirring. For cross‐linking, 2% (w/v) CaCl_2_ is added to the cooled mixture in a petri dish and left for 24 h. Excess CaCl_2_ is removed, and the hydrogels are washed three times with double‐distilled water. To enhance stability, a freeze–thaw cycle (freezing at −20°C for 18 h, thawing at room temperature for 6 h, repeated 3 times) is applied. Finally, the samples are freeze‐dried for 24 h to obtain a porous, stable hydrogel.

### 2.7. Hydrogel Characterization

#### 2.7.1. Hydrogel Swelling Ratio

The swelling behavior of the hydrogel was measured by a gravimetric method. Synthesized hydrogel samples were prepared in 24‐well plates and processed by cross‐linking, freeze–thawing, and freeze‐drying (Figure 3(b)). Equal initial dry weights (*W*_*d*_) of the samples were ensured (Figure 3(c)). The hydrogels were placed in a sieve immersed in water to swell. At set time intervals, samples were removed, surface water gently blotted, weighed, and returned to the solution. Three samples were tested independently. The swelling percentage (*S*%) was calculated using [20]:

(2) S%=Ww−WdWd×100,

where *W*_*w*_ represents the weight of the swollen hydrogel, and *W*_*d*_ is the initial dry weight of the sample.

#### 2.7.2. Hydrogel Porosity Measurements

The porosity of the hydrogels was assessed through the liquid displacement method. The samples (Figure 3(b)) were fully immersed in ethanol until saturation was achieved, after which they were weighed again. This procedure was performed three times to ensure accuracy. The porosity of the hydrogels was then calculated using the following equation (equation (3)) [21]:

(3) Porosity%=W2−W1ρV×100,

here, *W*_1_ and *W*_2_ represent the weights of the hydrogels before and after immersion in absolute ethanol, respectively. The parameter *ρ* denotes the density of absolute ethanol (0.785 g/cm^3^), while *V* refers to the volume of the sample prior to immersion.

#### 2.7.3. ATR‐FTIR Analysis

The ATR‐FTIR spectra of PSH and PSH/ZnO NPs‐CH freeze‐dried hydrogels were recorded using a Bruker ATR‐FTIR spectrometer to identify chemical constituents and bond configurations. The spectra were collected in the 400–4000 cm^−1^ range at room temperature with a resolution of 4 cm^−1^, averaging 32 scans per sample. A diamond ATR crystal was used, and the freeze‐dried hydrogel samples were placed directly on the crystal without additional preparation.

#### 2.7.4. Morphology Analysis

The microstructure of the hydrogels was examined using a field‐emission scanning electron microscope (FE‐SEM, TESCAN MIRA3, TESCAN, Czech Republic). Freeze‐dried hydrogel samples were cut into 1 × 1 cm^2^ sections and sputter‐coated with a thin layer of gold to enhance electrical conductivity. FE‐SEM imaging was performed to observe the porous structure of the hydrogels and to evaluate the distribution of ZnO NPs‐CH inside and on the surface of the pores in the PSH/ZnO NPs‐CH hydrogel.

Elemental composition and distribution of ZnO NPs‐CH within the hydrogel matrix were further confirmed by energy‐dispersive X‐ray spectroscopy (EDX) coupled with FE‐SEM.

#### 2.7.5. Contact Angle (CA)

The CA test evaluates the wettability and interaction of hydrogel membranes with liquids by measuring the angle formed where a liquid droplet meets the hydrogel surface. This measurement provides key information about the hydrogel’s hydrophilic or hydrophobic nature. For skin‐contact materials, balancing hydrophilicity and hydrophobicity is important [22]. Hydrogels with a CA below 90° are considered hydrophilic [23].

The wettability of the freeze‐dried PSH/ZnO NPs‐CH hydrogel membranes (1 × 1 cm^2^) was assessed by static CA measurements using a goniometer (CAG‐20 Jikan, Jikan Company, Iran). Droplets of deionized water with a volume of 5 μL were carefully deposited onto the hydrogel surface using a microsyringe at a constant injection rate of 0.2 μL/s. The CA was immediately recorded 5 s after deposition to minimize errors caused by rapid water absorption into the hydrogel network. The experiments were performed at ambient laboratory conditions (25°C, 33% relative humidity, and 88 kPa pressure). Both the advancing and receding angles were evaluated by gradually increasing or withdrawing the droplet volume, while the static angle was considered as the main reported value. Angles were analyzed using Jika Assistant software (Version 3.5), and in cases of image noise, supplementary analysis was performed using ImageJ software.

### 2.8. Evaluation of Antibacterial Activity

The antibacterial potential of the hydrogels was evaluated using the agar well diffusion method according to CLSI/ISO terminology. Bacterial strains were obtained from the Iranian Research Organization for Science and Technology (IROST) in Tehran, Iran. The selected strains included the Gram‐positive bacterium *S. aureus* (ATCC 25923) and the Gram‐negative bacterium *E. coli* (ATCC 25922). The bacterial inoculum was standardized to 0.5 McFarland (∼1.5 × 10^8^ CFU/mL) and confirmed by optical density measurement. Nutrient agar was used as the culture medium. It was dissolved in double‐distilled water, autoclaved at 121°C for 15 min at 15 psi, and then cooled before 25 mL of the medium was poured into each petri dish, achieving an agar depth of approximately 4 mm. An inoculum suspension (0.1 mL) was uniformly spread onto the surface of the agar medium using a sterile L‐shaped glass rod. To ensure even bacterial growth, the plates were streaked in one direction, rotated 90°, and streaked again; this was repeated three times. The plates were then allowed to dry for approximately five minutes. Four wells (6 mm in diameter) were aseptically created in each agar plate using a sterile cork borer. Intact hydrogel samples (PSH and PSH/ZnO NPs‐CH) were added to the wells at a volume of 50 μL per well, while the standard antibiotic gentamicin (positive control) was applied as a disc. The plates were incubated at 37°C for 24 h. All experiments were performed in triplicate (*n* = 3), and the diameter of the inhibition zones was measured using ImageJ software and reported as mean ± SD.

### 2.9. *In Vitro* Release Study

To determine the Zn loading per unit area of the dressings, square cutouts of 1 × 1 cm^2^ were prepared (*n* = 3). Each sample was completely digested in 5 mL of concentrated nitric acid (HNO_3_, trace metal grade) with gentle heating (90°C) until a clear solution was obtained. The digests were diluted to 25 mL with deionized water and analyzed for Zn content by flame atomic absorption spectrophotometry (Shimadzu AA‐680, air‐acetylene flame, *λ* = 213.9 nm). Calibration curves were obtained from standard Zn^2+^ solutions (Merck) in the range of 1–10 mg/L (*R* ^2^ > 0.99). Blank digestions were carried out in parallel. The Zn content per dressing area (μg/cm^2^) was calculated as:

(4) Zn contentμg/cm2=C×VA,

where *C* is the measured Zn concentration (μg/mL), *V* the final digest volume (mL), and *A* the dressing area (cm^2^).

To evaluate Zn release, dressing cutouts (1 × 1 cm^2^, *n* = 3) were immersed in 10 mL phosphate‐buffered saline (PBS, pH 7.4 and 5.8) at 37°C in sealed glass vials and maintained under gentle shaking (100 rpm). At predetermined time intervals (2, 12, 24, and 48 h), 1 mL aliquots were withdrawn and immediately replaced with equal volumes of fresh PBS. The samples were acidified with 1% HNO_3_ and analyzed for Zn by flame‐AAS as described above.

### 2.10. *In Vivo* Animal Studies

The experimental procedures, including the use of rats, were evaluated and approved by the Animal Ethics Committee of Razi University. This study adhered to the ethical guidelines outlined in the *Guide for the Care and Use of Laboratory Animals* by the Iran National Committee for Ethics in Biomedical Research (Approval Number: IR.RAZI.AEC.1403.055). All procedures were reported following the ARRIVE guidelines, including daily dressing and hydrogel application, ensuring full compliance with ARRIVE 2.0 checklist recommendations.

#### 2.10.1. Wound Contraction Studies

Twenty adult male Wistar rats (200–250 g) were obtained from the Animal House of the Faculty of Pharmacy at Kermanshah University of Medical Sciences, Iran. The sample size (*n* = 5 per group) was determined based on a priori power analysis (*α* = 0.05, power = 0.8) using data from previous wound‐healing studies, which indicated that five animals per group would be sufficient to detect a 20% difference in wound closure rates.

Rats fasted overnight before anesthesia (ketamine 80 mg/kg and xylazine 20 mg/kg, intramuscular). Post‐operative analgesia was provided with meloxicam (1 mg/kg, subcutaneous) immediately after surgery and then once daily for 3 consecutive days to minimize pain [24]. After shaving and disinfecting the dorsal area, a 1.5 cm circular full‐thickness clean surgical wound was created on each rat. Treatments were applied once daily for 14 days starting from the day of surgery (Day 0) [25].

Animals were randomly allocated to four groups (*n* = 5 each) using a computer‐generated random number sequence:

• Group I (negative control): treated with double‐distilled water.
• Group II (positive control): treated with 1% phenytoin topical gel.
• Group III: treated with PSH hydrogel.
• Group IV: treated with PSH/ZnO NPs‐CH hydrogel.

**Figure 4:**
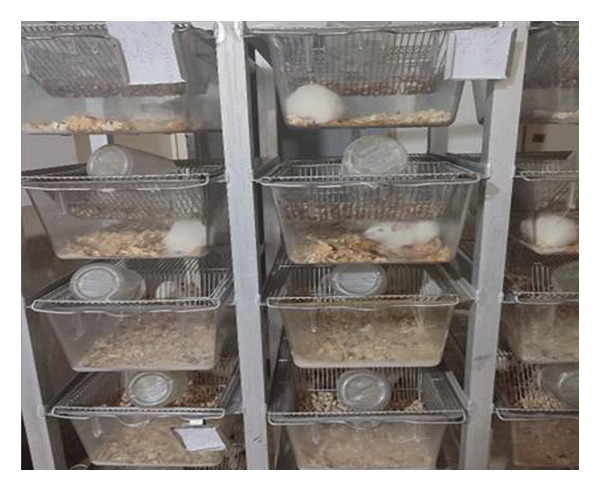
Housing and maintenance of laboratory rats in separate cages under controlled conditions for experimental studies.

Animals were housed individually under controlled conditions (12 h light/dark cycle, 22 ± 3°C, 45%–50% humidity) with free access to food and water (see Figure 4). Exclusion criteria, including signs of infection or abnormal behavior, were predefined; however, no animals met these criteria during the study, and no adverse effects related to hydrogel application were observed. Circular hydrogel dressings with a diameter of 2 cm were prepared and applied directly to cover the wound area. Each hydrogel sheet was retained in situ and secured using a sterile mesh adhesive tape (Omnifix®, Hartmann, Germany) to prevent displacement. This type of adhesive mesh tape was selected because of its breathability, flexibility, and hypoallergenic properties, which ensured stable fixation of the hydrogel without impairing wound healing or causing skin irritation. Dressings were replaced daily with freshly prepared hydrogels.

#### 2.10.2. Percentage of Wound Healing

Wounds were photographed using a digital camera under standardized lighting conditions, with a millimeter scale placed adjacent to the wound to provide size calibration. Wound diameter was measured with a ruler on Days 0, 3, 7, 9, and 14. In addition, wound area was determined using ImageJ software (NIH, Bethesda, MD, USA) by manually contouring the wound margins to ensure accurate measurement of wound contraction. Since wounds were created manually using surgical scissors, minor variations in the initial wound size were unavoidable. Therefore, the exact wound area on Day 0 (*W*_*A*0_) was measured individually for each animal using digital photographs calibrated with a millimeter scale and analyzed with ImageJ software. This baseline value was then used for normalization of wound contraction calculations to eliminate the effect of inter‐animal variability in initial wound size. Assessors who measured wound healing were blinded to treatment groups. The percentage of wound closure (*W*_*C*_) was calculated using the following equation:

(5) % of WC=WA0−WAnWA0×100.

In this equation, *W*_*C*_ represents the percentage of wound closure, *W*_*A* *n*_ is the wound area on day *n* (where *n* = 3, 7, 9, or 14), *W*_*A*0_ is the initial wound area on Day 0.

#### 2.10.3. Histological Observation

On Days 7 and 14, one rat from each group was euthanized using an overdose of ketamine and xylazine. Skin tissue samples from the wound sites were collected, fixed in 10% neutral‐buffered formalin, and gradually dehydrated using increasing concentrations of ethanol. The samples were then embedded in paraffin, sectioned into 5–6 μm slices, stained with hematoxylin and eosin (H&E), and examined under a light microscope for histological analysis.

### 2.11. Statistical Analysis

Statistical analysis was conducted using SPSS software (Version 16.0, IBM, Chicago, IL, USA). Data were analyzed by one‐way analysis of variance (ANOVA), followed by Tukey’s​ post hoc test for multiple comparisons. Results are presented as mean ± SD from independent experiments. Most assays were performed in triplicate (*n* = 3), while the *in vivo* wound healing experiments were carried out in quintuplicate (*n* = 5) to increase reliability. Significant differences among groups are indicated using different letters or symbols, with exact *p* values reported in the figures and tables as applicable.

## 3. Results and Discussion

### 3.1. Characterization of NPs (ZnO NPs and ZnO NPs‐CH)

#### 3.1.1. UV–Vis Spectroscopy

**Figure 5:**
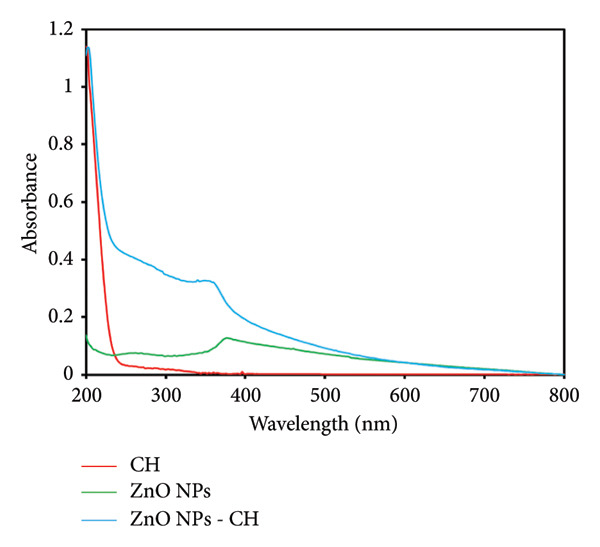
UV–vis absorption spectra of chitosan (CH), ZnO nanoparticles (ZnO NPs), and ZnO NPs–CH.

UV–vis spectroscopy is an effective technique for confirming the formation of green‐synthesized ZnO NPs and ZnO NPs‐CH. Figure 5 presents the UV–vis spectra of CH, ZnO NPs, and ZnO NPs‐CH samples at identical concentrations. The UV–vis absorption spectrum of CH did not exhibit any distinct absorption maxima within the 250–500 nm range, as the CH molecule lacks conjugated double bonds [26]. In contrast, ZnO NPs typically display a characteristic exciton peak wavelength range. The green‐synthesized ZnO NPs in this study demonstrated an absorption maximum around 365 nm, which corresponds to their intrinsic band‐gap absorption, attributed to electron transitions from the valence band to the conduction band (O_2p_ ⟶ Zn_3d_) [27]. Additionally, the absorption band of ZnO NPs coated with CH appears as a combination of the absorption bands of both uncoated ZnO NPs and CH. This spectral feature confirms the successful coating of ZnO NPs with CH, indicating an interaction between the NPs and the CH matrix [28].

#### 3.1.2. Morphological Analysis

The morphology of green‐synthesized ZnO NPs was examined using SEM imaging. As illustrated in Figure 6(a), the ZnO NPs exhibit a quasi‐spherical shape with a slight tendency to aggregate, likely due to the sample preparation process. However, they remain well dispersed. In contrast, ZnO NPs‐CH display a distinct shell‐like and sheet‐like morphology, as shown in Figure 6(b). This structural modification suggests that the CH layer significantly influences the morphology of ZnO NPs, indicating a strong interaction between ZnO and CH [28].

Figure 6SEM images of (a) ZnO NPs and (b) ZnO NPs‐CH.

**Figure:**
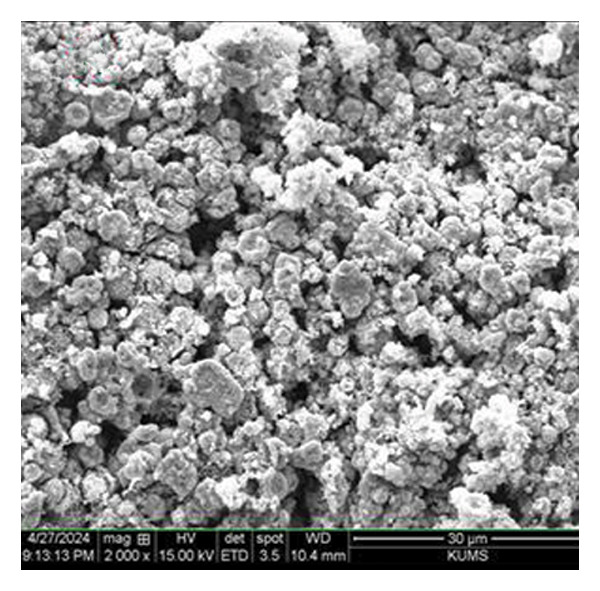
(a)

**Figure:**
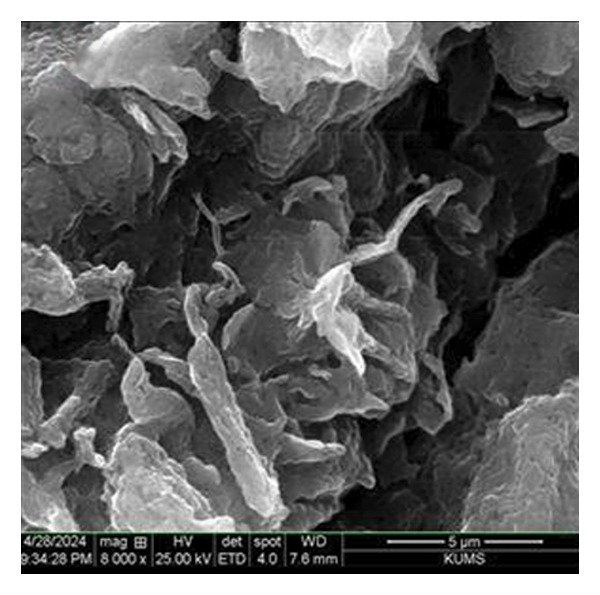
(b)

#### 3.1.3. Zeta Potential Analysis

Coating ZnO NPs with CH changes their zeta potential from −28 mV to −9 mV (Figure 7(a) and 7(b)), indicating successful surface modification through interaction between CH amino groups and ZnO surface groups [29]. This reduces the surface negative charge, improving compatibility with biological systems. The increased surface charge enhances electrostatic attraction to negatively charged bacterial membranes, promoting better adhesion and cellular penetration. Additionally, CH’s inherent antibacterial properties synergize with ZnO, boosting antimicrobial effects by disrupting membranes and inhibiting metabolism [28]. The NPs remain moderately stable due to their negative zeta potential [30].

Figure 7Zeta potential distribution of (a) ZnO NPs green‐synthesized using wild cherry shrub wood extract and (b) ZnO NPs‐CH.

**Figure:**
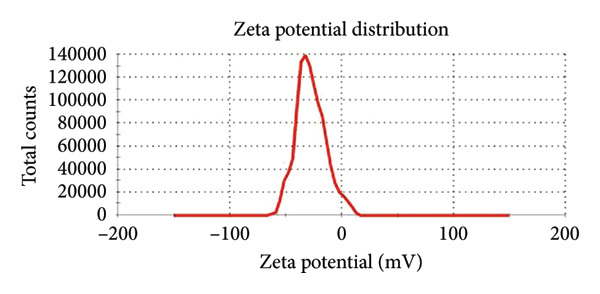
(a)

**Figure:**
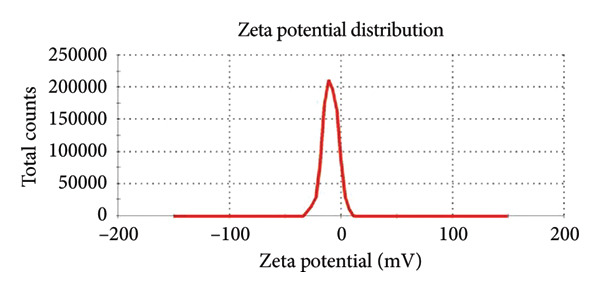
(b)

#### 3.1.4. TEM Images

The TEM images of the ZnO NPs‐CH confirm their successful encapsulation within the polymeric matrix (CH) (Figure 8(a)). The images reveal a strong interaction between the CH phase and the ZnO NPs, suggesting favorable electrostatic or hydrogen bonding interactions that contribute to their stability. The ZnO NPs predominantly exhibit a quasi‐spherical morphology, although some irregularly shaped particles are also observed. The average particle size was measured to be approximately 81.14 nm (Figure 8(b)), indicating a nanoscale distribution suitable for biomedical applications [28].

Figure 8(a) TEM image of ZnO NPs coated with CH, showing the morphology and distribution of ZnO NPs within the CH matrix. Note: The left TEM image has been digitally adjusted for clarity; therefore, it does not display the original instrument magnification or scale bar. (b) Grain size distribution histogram of ZnO NPs coated with CH.

**Figure:**
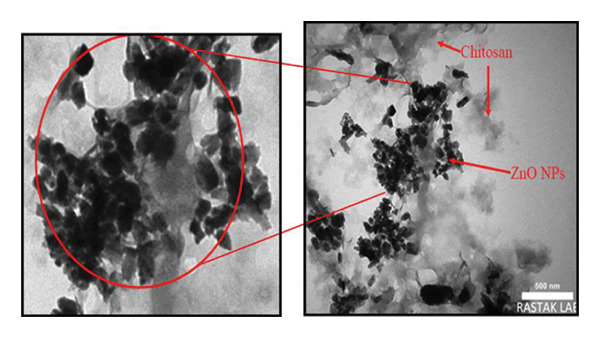
(a)

**Figure:**
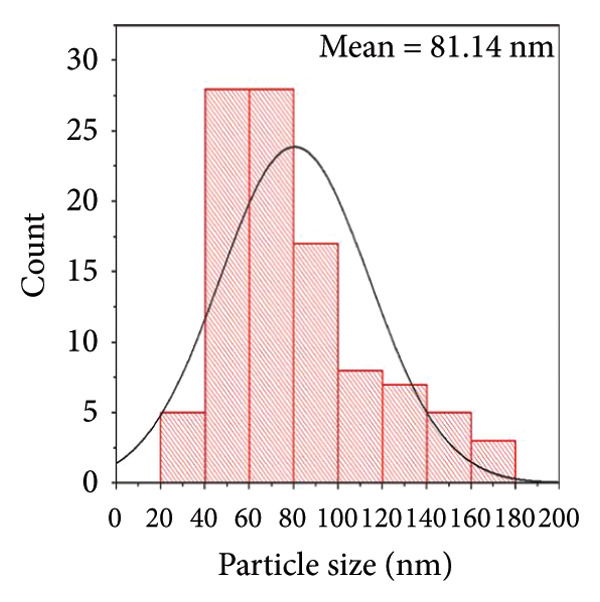
(b)

### 3.2. Characterization of Hydrogels

#### 3.2.1. Investigation of Morphology and Structure of Hydrogels Using FE‐SEM

**Figure 9:**
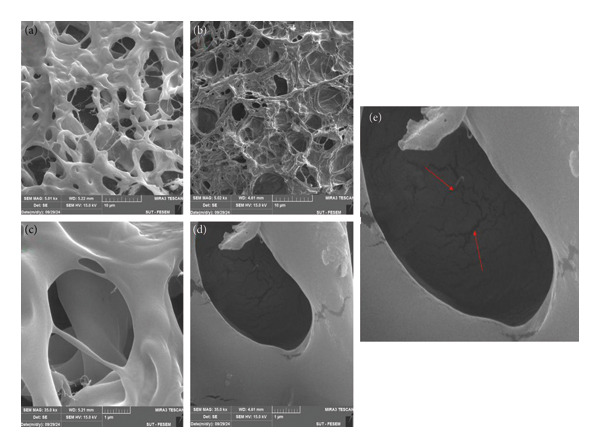
FE‐SEM images of PSH hydrogel and PSH/ZnO NPs‐CH hydrogel at different magnifications. (a) and (b) show the porous structure of the PSH hydrogel without and with ZnO NPs‐CH, respectively, (c) is a higher‐magnification image of the porous structure of PSH hydrogel without ZnO NPs‐CH, (d) is a high‐magnification image showing the internal distribution of ZnO NPs‐CH within the hydrogel matrix, and (e) is a close‐up image highlighting the detailed internal distribution of ZnO NPs‐CH within the hydrogel matrix, indicated by red arrows.

FE‐SEM was used to examine the shape, surface morphology, and porosity of the hydrogel matrix, while EDX provided elemental analysis. FE‐SEM images at various magnifications of the two hydrogels prepared by freeze‐drying are shown in Figure 9. The morphologies of PSH and PSH/ZnO NPs‐CH hydrogels are presented in Figures 9(a) and 9(b), respectively. Both hydrogels exhibited 3D interconnected porous networks facilitating the penetration and diffusion of water vapor and gases, aiding wound healing [20]. The hydrogel without ZnO NPs‐CH showed a smooth morphology, while ZnO NPs‐CH were homogeneously distributed on the PSH surface (Figure 9(b)). At higher magnifications, the porous structure coated with ZnO NPs‐CH is more evident (visible protrusions and particle‐like features on the PSH surface) [31]. This morphology likely results from interactions between ZnO NPs‐CH and PSH, contributing to the denser structure in the PSH/ZnO NPs‐CH hydrogel. Additionally, Figure 9(b) shows that PSH/ZnO NPs‐CH has a tighter structure compared to PSH.

Additionally, EDX analysis (Figures 10(a) and 10(b)) acknowledged C, O, Na, Ca, Cl, and N in the PSH and PSH/ZnO NPs‐CH hydrogels, which related to the PVA, SA polymers, and honey. The presence of Ca confirms the successful interaction between the polymeric chains and the cross‐linking agent [32]. The presence of Zn (3.90 wt%), along with the increased C content (from 37.73 to 41.74 wt%) and N content (from 1.02 to 2.15 wt%), correlates with the incorporation of ZnO NPs‐CH in the PSH hydrogel.

Figure 10EDX analysis results for the (a) PSH hydrogel and (b) PSH/ZnO NPs‐CH hydrogel, demonstrating the elemental composition and the presence of ZnO NPs‐CH in the PSH hydrogel structure.

**Figure:**
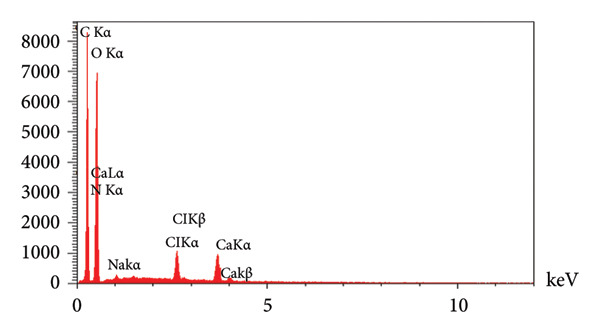
(a)

**Figure:**
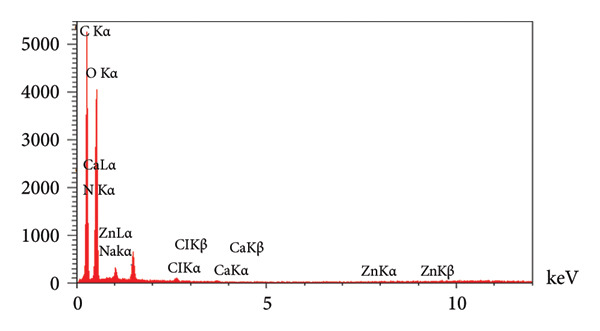
(b)

#### 3.2.2. ATR‐FTIR Analysis of Hydrogel

**Figure 11:**
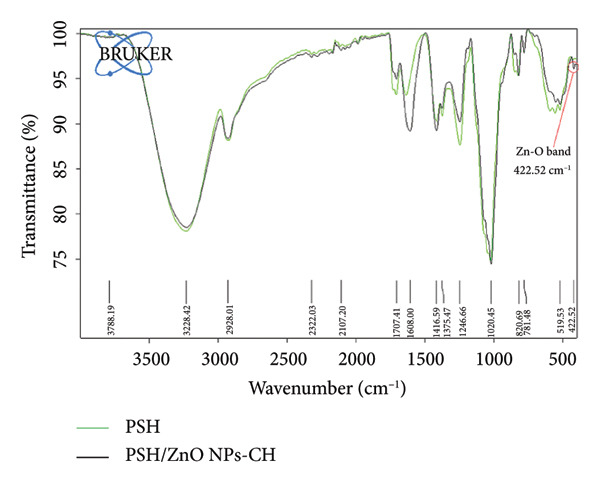
FTIR spectra of PSH hydrogel (green) and PSH/ZnO NPs‐CH hydrogel (black) presented in the same panel for direct comparison. The appearance of the Zn‐O stretching vibration at 423 cm^−1^ in the PSH/ZnO NPs‐CH spectrum confirms the successful incorporation of ZnO NPs.

The PSH and PSH/ZnO NPs‐CH polymeric hydrogels were analyzed as thin films to identify functional groups using ATR‐FTIR analysis. As shown in Figure 11, the band at 3236 cm^−1^ corresponds to the hydroxyl group (‐OH) in SA, PVA, and honey [33, 34]. The band at 1246 cm^−1^ is associated with the C‐O‐C stretching in SA and honey [35, 36]. Furthermore, the band at 1024 cm^−1^ is attributed to the aldehyde (CHO) group in SA, PVA, and honey. The asymmetric peaks at 1637 cm^−1^ and 1416 cm^−1^ correspond to the sodium carboxylate group [37, 38]. The band at 1709 cm^−1^ represents the carbonyl group (C=O) in SA and honey and is also related to the tautomerization of PVA [37].

In Figure 11, it is evident that the PSH/ZnO NPs‐CH polymeric hydrogel shows similar bands, with some changes in position and intensity. The broadband at 3228 cm^−1^ corresponds to the hydroxyl group (‐OH) [18, 39]. The amine group (N‐H) from CH also appears in the region 3500–3300 cm^−1^, overlapping with the broad peak of the ‐OH group of SA, PVA, and honey [40]. Additionally, the absorption band observed at 423 cm^−1^ is attributed to the stretching vibrations of ZnO [41, 42].

#### 3.2.3. Hydrogel Swelling Ratio

Swelling capacity is critical in biomedical applications, especially wound dressings, as it helps absorb exudates, maintain moisture, and promote healing [11]. Table 1 illustrates the swelling behavior of PSH and PSH/ZnO NPs‐CH hydrogels over time. The PSH hydrogel exhibited a rapid initial swelling (314.26 ± 1.33% in the first min), increasing to a maximum of 403.97 ± 1.07% at 120 min, followed by minor fluctuations likely due to water absorption‐release dynamics and polymer network rearrangement. In contrast, the PSH/ZnO NPs‐CH hydrogel showed a slower, more stable swelling behavior, reaching 302.19 ± 0.50 at 180 min and stabilizing around 281.46 ± 0.50% at 360 min. The more consistent swelling is attributed to the structural reinforcement by ZnO NPs coated with CH, which enhances mechanical stability and reduces fluctuations. Initial weight loss in both hydrogels may result from the leaching of soluble components like honey. According to de la Mora‐López et al. [33], the high solubility of honey contributes to its degradation and a temporary reduction in swelling capacity. Subsequent reabsorption is likely due to polymer rearrangement, osmotic effects, or new interactions among polymer chains. Osmotically active honey constituents may also induce fluid shifts, causing weight variations. Statistical analysis (one‐way ANOVA, *p* < 001) demonstrated that the differences in swelling ratios between PSH and PSH/ZnO NPs‐CH hydrogels at their respective equilibrium states are statistically significant. This indicates that incorporation of ZnO NPs‐CH not only reduces the overall swelling but also stabilizes water uptake. The observed differences can be attributed to material composition: PSH hydrogels, containing a higher fraction of soluble honey, tend to lose part of this component during swelling, leading to temporary decreases and subsequent fluctuations [43]. In contrast, the denser polymer network reinforced with ZnO NPs‐CH limits leaching, minimizes osmotic disturbances, and results in a more stable swelling profile. Consistent with previous reports where ZnO‐based nanocomposites reduced porosity and acted as crosslinking agents in polymer matrices, thereby lowering and stabilizing swelling ratios [44], our findings confirm that ZnO NPs‐CH reinforcement provides a more stable swelling behavior suitable for wound dressing applications.

**Table 1:** Swelling ratio (%) of hydrogel and hydrogel containing NPs at different time intervals.

Time (min)	Hydrogel (%)	Hydrogel + NPs (%)
0	0.00 ± 0.00^a^	0.00 ± 0.00^a^
1	**314.26 ± 1.33** ^**l**^	198.29 ± 0.95^e^
2	343.49 ± 1.29^mn^	195.61 ± 0.65^e^
3	362.77 ± 2.38^p^	196.42 ± 0.72^e^
4	344.12 ± 0.70^mn^	170.73 ± 0.10^c^
5	348.22 ± 0.60^o^	169.02 ± 0.34^c^
10	345.93 ± 0.51^no^	160.73 ± 0.94^b^
15	362.07 ± 0.80^p^	187.89 ± 0.67^d^
20	341.61 ± 1.29^m^	189.11 ± 1.19^d^
25	382.67 ± 1.10^r^	197.89 ± 1.17^e^
30	387.06 ± 0.84^s^	220.49 ± 0.15^f^
60	392.21 ± 0.72^t^	236.91 ± 0.56^g^
120	**403.97 ± 1.07** ^**v**^	274.47 ± 0.45^h^
180	386.64 ± 2.22^s^	**302.19 ± 0.50** ^**k**^
240	399.51 ± 1.12^u^	284.15 ± 0.69^j^
300	387.13 ± 2.12^s^	280.16 ± 0.71^i^
360	366.32 ± 2.10^q^	**281.46 ± 0.50** ^**ij**^

*Note:* Values are presented as mean ± SD of three independent replicates (*n* = 3). Data met the assumptions of ANOVA (normality and homogeneity of variance). Different letters (a–v) indicate statistically significant differences among groups at each time point, as determined by one‐way ANOVA followed by Tukey’s post hoc test (*p* < 0.001). Bold values indicate the maximum swelling ratio observed for each sample.

The swelling kinetics of both pristine hydrogel and hydrogel containing NPs were analyzed using pseudo‐first‐order (PFO) and pseudo‐second‐order (PSO) kinetic models. As summarized in Table 2, both models exhibited good correlation with the experimental data (*R* ^2^ > 0.94). For the neat hydrogel, the equilibrium swelling ratio (*Q*∞) reached approximately 410%, whereas incorporation of NPs reduced this value to ∼310%. The rate constants (*k*_1_ for PFO and *k*_2_ for PSO) also decreased in the presence of NPs, indicating a slower swelling process. Notably, the PSO model provided a slightly better fit (higher *R* ^2^ values), suggesting that the swelling behavior is more accurately described by a second‐order kinetic mechanism, where polymer chain relaxation and network reorganization play a significant role. These findings imply that NPs restrict the free volume within the hydrogel matrix, thereby hindering water uptake and reducing the overall swelling capacity.

**Table 2:** Kinetic parameters for swelling behavior of hydrogel and hydrogel + NPs.

Sample	Model	*k* (min^−1^/min·%^−1^)	*Q*∞ (swelling ratio, %)	*R* ^2^
Hydrogel	PFO	0.025	410	0.96
Hydrogel	PSO	1.2 × 10^−4^	405	0.97
Hydrogel + NPs	PFO	0.018	310	0.94
Hydrogel + NPs	PSO	1.1 × 10^−4^	315	0.96

#### 3.2.4. Hydrogel Porosity Measurements

The porous structure of hydrogels facilitates the absorption of large amounts of wound exudates while also promoting the distribution of nutrients. This characteristic creates an optimal environment for cell adhesion and proliferation, supporting the wound healing process [20]. Here, the hydrogel porosity decreases from 58.38 ± 1.64% to 42.63 ± 1.78% with the addition of ZnO NPs‐CH during the preparation. This may be referred to as ZnO NPs‐CH filling the pores in the PSH hydrogel and making it more compact [45]. This pattern was further validated by the FE‐SEM micrographs presented in Figure 9. On the other hand, as reported by Şalva and Servín de la Mora, an increase in honey concentration within the hydrogel leads to a decrease in pore size. Despite this reduction, the data suggest that the hydrogel retains pores suitable for wound healing. Furthermore, the presence of ZnO NPs‐CH and honey enhances its wound healing properties [33, 46].

The observed decrease in porosity directly correlates with the swelling behavior reported in Section 3.2.3. The PSH/ZnO NPs‐CH hydrogel, having a denser and less porous structure, exhibits lower but more stable water absorption compared to PSH. In other words, the presence of ZnO NPs‐CH reduces maximum swelling while stabilizing the absorption profile. Nevertheless, the hydrogel maintains sufficient porosity for fluid uptake and nutrient transport, essential for supporting wound healing. Thus, the porosity measurements and FE‐SEM observations explain the differences in swelling behavior between the hydrogels.

#### 3.2.5. CA Measurements

**Figure 12:**
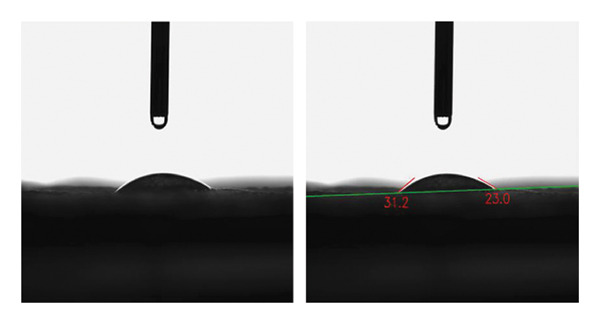
Contact angle (CA) analysis of PSH/ZnO NPs‐CH hydrogel.

CA in hydrogels is a key parameter indicating surface wettability and hydrophilicity or hydrophobicity. A CA less than 90° shows a hydrophilic surface, while greater than 90° indicates hydrophobicity. Adding honey reduces CA, increasing hydrophilicity [15]. However, excessively low CA can cause over‐absorption of water, risking mechanical weakening or bacterial growth [11, 47]. Thus, an optimal CA balances hydrophilicity, mechanical stability, and moisture control. The PSH/ZnO NPs‐CH hydrogel exhibits an average CA of 37.5 ± 4.70°, indicating good hydrophilicity suitable for antibacterial and wound healing applications (Figure 12). This hydrophilicity improves fluid interaction, maintains wound moisture, accelerates healing, and, combined with ZnO NPs‐CH’s strong antibacterial effects, enhances diffusion of agents and oxygen/nutrient exchange, promoting faster wound repair.

### 3.3. Antibacterial Assessments

**Figure 13:**
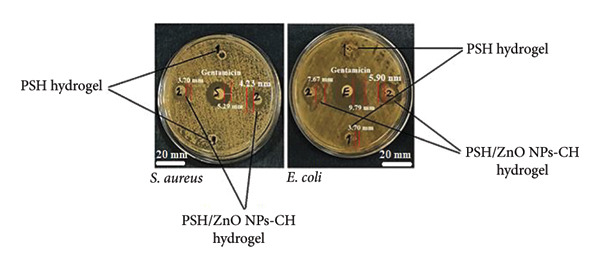
Antibacterial activity of PSH hydrogel and PSH/ZnO NPs‐CH hydrogel against *S. aureus* and *E. coli*. The inhibition zone diameters indicate enhanced antibacterial performance in the presence of ZnO NPs‐CH compared to the blank hydrogel. Representative antibacterial plate showing two replicates; quantitative results are presented as mean ± SD (*n* = 3) in Table 3. Gentamicin is used as a positive control.

The antibacterial activity of PSH and PSH/ZnO NPs‐CH hydrogels was evaluated against *E. coli* and *S. aureus* using the disc diffusion method. The PSH hydrogel showed no inhibition against *S. aureus*, whereas incorporation of green‐synthesized ZnO NPs coated with CH significantly enhanced antibacterial performance. The PSH/ZnO NPs‐CH hydrogel exhibited inhibition zones of 6.57 ± 0.78 mm for *E. coli* and 3.98 ± 0.22 mm for *S. aureus* (Figure 13 and Table 3), indicating moderate yet notable antibacterial activity. Compared to the standard antibiotic gentamicin (9.84 ± 0.20 mm for *E. coli* and 5.26 ± 0.09 mm for *S. aureus*) [48], the hydrogel showed relatively lower efficacy, but it significantly outperformed previous collagen‐based hydrogels containing silver NPs (Col I/AgNPs), which demonstrated only 1.92 mm and 2.01 mm inhibition zones against *E. coli* and *S. aureus*, respectively [31]. The enhanced performance of ZnO NPs‐CH may be attributed to their ability to generate reactive oxygen species (ROS) such as superoxide anions (·O_2_^−^), hydroxyl radicals (·OH), and hydrogen peroxide (H_2_O_2_), which disrupt bacterial membranes [49]. Additionally, honey’s inherent antibacterial properties due to its high sugar content, low pH, hydrogen peroxide generation, and flavonoids further contribute to the overall antimicrobial effect [16, 50]. These results support the potential of PSH/ZnO NPs‐CH hydrogel as a promising antibacterial wound dressing with effectiveness approaching that of conventional antibiotics.

**Table 3:** Antibacterial results of PSH and PSH/ZnO NPs‐CH hydrogel against *S. aureus* and *E. coli* based on the agar diffusion method.

	Zone of inhibition (mm)	
	*S. aureus*	*E. coli*
Gentamicin	5.26 ± 0.09^c^	9.84 ± 0.20^e^
PSH hydrogel	0.00 ± 0.00^a^	3.77 ± 0.09^b^
PSH/ZnO NPs‐CH hydrogel	3.98 ± 0.22^b^	6.57 ± 0.78^d^

*Note:* Values are presented as mean ± SD of three independent replicates (*n* = 3). Different letters indicate statistically significant differences among groups, as determined by one‐way ANOVA followed by Tukey’s post hoc test (*p* < 0.001).

### 3.4. *In Vitro* Release Study

**Figure 14:**
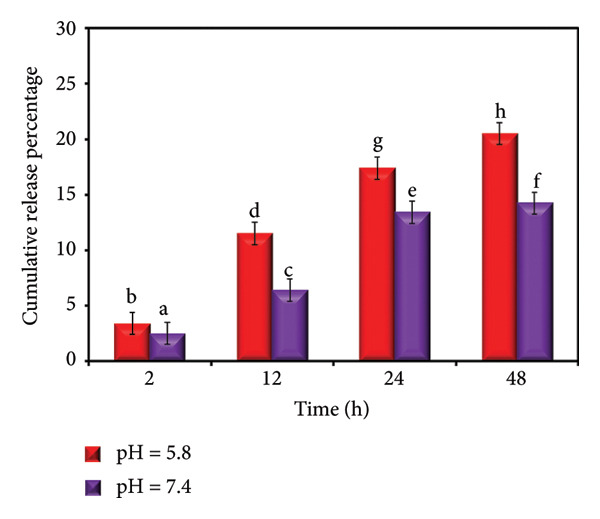
The release profile of ZnO NPs from the PSH/ZnO NPs‐CH hydrogel at two different pHs 7.4 and 5.8. Values are presented as mean ± SD of three independent replicates (*n* = 3). Different letters indicate statistically significant differences among groups, as determined by one‐way ANOVA followed by Tukey’s post hoc test (*p* < 0.001).

The *in vitro* release profile of ZnO NPs from the hydrogel formulations at two different pH conditions (PBS, pH 7.4 and 5.8) demonstrated a distinct pH‐responsive behavior (Figure 14). The release followed a biphasic trend with an initial burst within the first 12 h, followed by a slower and sustained release phase up to 48 h. The total Zn loading of the dressing was 184.07 ± 2.67 μg/cm^2^. At pH 5.8, Zn release reached 6.31 ± 0.23, 21.10 ± 0.47, 32.03 ± 0.66, and 37.73 ± 0.72 μg/cm^2^ at 2, 12, 24, and 48 h, corresponding to approximately 3.4%–20.5% of the initial loading. At pH 7.4, the respective release values were 4.54 ± 0.25, 11.84 ± 0.37, 24.67 ± 0.51, and 26.20 ± 0.52 μg/cm^2^, accounting for approximately 2.5%–14.2% of the loading. Overall, release under mildly acidic conditions (pH 5.8, mimicking inflamed/wounded tissue) was faster than at physiological pH 7.4 [49].

This enhanced release under acidic conditions can be attributed to the increased solubility of ZnO NPs and weakened polymer‐ionic interactions in the hydrogel matrix at lower pH, which facilitates NP diffusion. Such pH‐dependent release behavior is advantageous for wound healing applications: The normal physiological environment maintains a slower baseline release, preventing rapid depletion of the active agent, while the acidic milieu of infected or inflamed wounds promotes accelerated release, enhancing antibacterial efficacy and supporting tissue regeneration.

Importantly, the Zn loading in our dressing is over 10 times lower than the dermal exposure levels tested by Hansen et al., where topical application of 2000 μg/cm^2^ nanostructured ZnO (NM‐111) in rats showed no signs of acute dermal toxicity or systemic Zn absorption. This comparison suggests that the Zn content and release profile in our system likely fall within a safe dermal exposure range. Moreover, even these relatively low local Zn concentrations are expected to potentially provide antibacterial activity and promote wound healing by releasing Zn^2+^ ions locally at the wound site, without significant systemic absorption, consistent with previous findings on the safety and efficacy of nanostructured ZnO in topical applications [51].

### 3.5. *In Vivo* Wound Healing Studies

**Figure 15:**
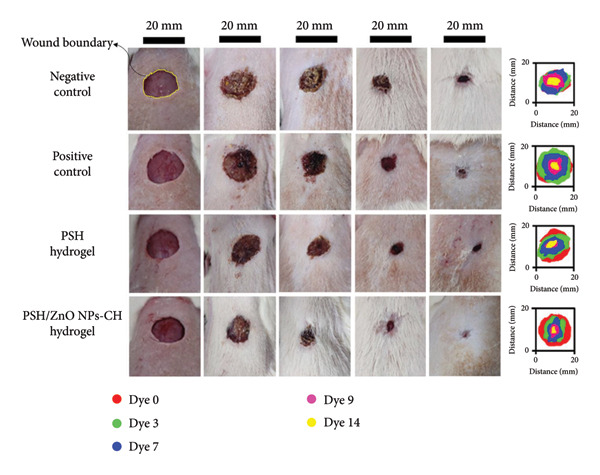
Representative images of wound healing progression over 14 days in different treatment groups, including the negative control, positive control, PSH hydrogel, and PSH‐ZnO NPs‐CH hydrogel. The images show wound closure on Days 0, 3, 7, 9, and 14. Wounds were photographed under standardized lighting conditions with a millimeter scale for calibration. The wound area was delineated (contoured) and measured using ImageJ software, and a healing assessment was performed by blinded evaluators. The rightmost column presents graphical representations of wound area reduction over time, with different colors corresponding to specific days.

**Figure 16:**
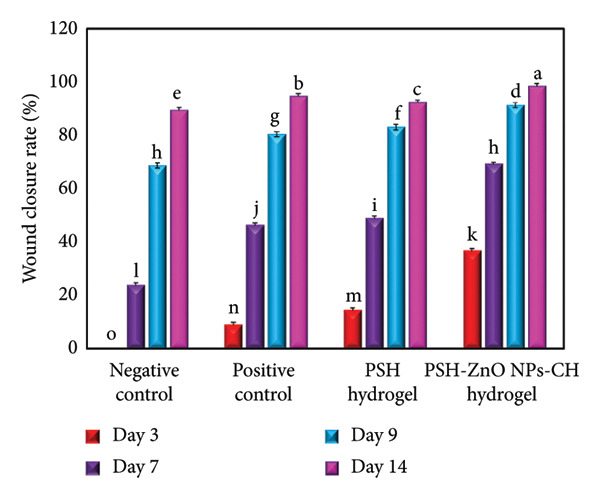
Wound closure rate (%) over 14 days for different treatment groups, including the negative control, positive control, PSH hydrogel, and PSH‐ZnO NPs‐CH hydrogel. Data were recorded on Days 3, 7, 9, and 14. Values are expressed as mean ± SD of five replicates (*n* = 5). Data met the assumptions of ANOVA (normality and homogeneity of variance). Different letters indicate statistically significant differences among groups at each time point, as determined by one‐way ANOVA followed by Tukey’s post hoc test (*p* < 0.001).

In this study, the wound healing efficiency of different formulations was macroscopically evaluated over a 14‐day period. No mortality was observed during the experiment, and the rats showed normal growth. Photographs of wounds from all groups are shown in Figure 15, the raw mean wound area (mm^2^) ± SD at each time point is summarized in Table 4, and the wound healing percentages at Days 3, 7, 9, and 14 post‐wounding are presented in Figure 16 and Table 5. It should also be noted that in cases where the rats’ hair had grown and interfered with imaging and precise measurement, shaving was performed. In the negative control group (Group I), which was treated only with double‐distilled water, wound healing was slow. On Day 3, no wound contraction was observed (−1.46 ± 0.40%), which increased to 23.62 ± 0.18% on Day 7, 68.70 ± 0.13% on Day 9, and finally 89.45 ± 0.01% on Day 14. Despite partial improvement, full wound closure was not achieved by the end of the treatment period. The positive control group (Group II), treated with 1% topical phenytoin gel, showed better results compared to the negative control. The wound contraction percentages were 8.87 ± 0.02%, 46.12 ± 0.11%, 80.44 ± 0.06%, and 94.58 ± 0.04% on Days 3, 7, 9, and 14, respectively. Although considerable healing was observed, minor signs of tissue damage persisted. In Group III (treated with PSH hydrogel), the wound healing process was better than in the negative control group. Wound contraction percentages were 14.22 ± 0.11% on Day 3, 48.75 ± 0.12% on Day 7, 83.03 ± 0.10% on Day 9, and 92.26 ± 0.07% on Day 14. These results indicate the intrinsic wound‐healing properties of the PSH hydrogel, which can be attributed to its bioactive components such as honey and the polymeric matrix. The best wound healing performance was observed in Group IV, treated with PSH hydrogel loaded with PSH/ZnO NPs‐CH. Wound contraction percentages in this group were 36.58 ± 0.10% on Day 3, 68.95 ± 0.06% on Day 7, 91.35 ± 0.06% on Day 9, and 98.41 ± 0.02% on Day 14, indicating nearly complete wound closure by the end of the study. These results highlight the high potential of the nanocomposite in accelerating tissue regeneration and reducing inflammation. Visual findings were consistent with numerical data. On Day 0, wound sizes were approximately the same across all groups, but differences became evident from Day 3 onward. Significant wound size reduction was seen in Group IV on Day 7, and near‐complete healing was observed by Day 9, whereas other groups, especially the negative control, still exhibited substantial unhealed areas. The accelerated healing in the PSH/ZnO NPs‐CH group is attributed to the combined effects of ZnO NPs and CH. ZnO NPs possess strong antimicrobial properties that prevent infections and provide a suitable environment for healing. CH is biocompatible, has anti‐inflammatory effects, and promotes cell proliferation and tissue regeneration [52–55]. Compared to phenytoin, a well‐known wound healing agent, the PSH/ZnO NPs‐CH hydrogel demonstrated superior performance in accelerating wound closure and tissue recovery. This suggests that this nanohydrogel formulation could serve as an effective alternative to conventional wound treatments.

**Table 4:** Wound area (mm^2^) of PSH and PSH/ZnO NPs‐CH hydrogels over 14 days.

	Negative control (mm^2^)	Positive control (mm^2^)	PSH hydrogel (mm^2^)	PSH‐ZnO NPs‐CH hydrogel (mm^2^)
Day 0	182.06 ± 0.72^p^	222.62 ± 0.35^s^	179.29 ± 0.28^o^	167.76 ± 0.39^n^
Day 3	184.73 ± 0.55^q^	202.88 ± 0.23^r^	153.78 ± 0.22^m^	106.40 ± 0.19^j^
Day 7	139.06 ± 0.57^l^	119.95 ± 0.43^k^	91.89 ± 0.28^i^	52.08 ± 0.09^g^
Day 9	56.99 ± 0.24^h^	43.54 ± 0.32^f^	30.41 ± 0.32^e^	14.51 ± 0.20^c^
Day 14	19.21 ± 0.07^d^	12.06 ± 0.13^b^	13.84 ± 0.21^c^	2.67 ± 0.06^a^

*Note:* Values are presented as mean ± SD of five independent replicates (*n* = 5). Data met the assumptions of ANOVA (normality and homogeneity of variance). Different letters indicate statistically significant differences among groups, as determined by one‐way ANOVA followed by Tukey’s post hoc test (*p* < 0.001).

**Table 5:** Wound closure rate (%) of PSH and PSH/ZnO NPs‐CH hydrogels over 14 days.

	Negative control	Positive control	PSH hydrogel	PSH‐ZnO NPs‐CH hydrogel
Day 3	−1.46 ± 0.40^o^	8.87 ± 0.02^n^	14.22 ± 0.11^m^	36.58 ± 0.10^k^
Day 7	23.62 ± 0.18^l^	46.12 ± 0.11^j^	48.75 ± 0.12^i^	68.95 ± 0.06^h^
Day 9	68.70 ± 0.13^h^	80.44 ± 0.06^g^	83.03 ± 0.10^f^	91.35 ± 0.06^d^
Day 14	89.45 ± 0.01^e^	94.58 ± 0.04^b^	92.26 ± 0.07^c^	98.41 ± 0.02^a^

*Note:* Values are presented as mean ± SD of five independent replicates (*n* = 5). Data met the assumptions of ANOVA (normality and homogeneity of variance). Different letters indicate statistically significant differences among groups, as determined by one‐way ANOVA followed by Tukey’s post hoc test (*p* < 0.001).

### 3.6. Histopathological Studies

This section presents a comparative histological evaluation of wound healing in different experimental groups using H&E staining at 40× and 400× magnifications. Normal skin structure consists of a thick epidermis and dermis, housing appendages, diverse cell types, and primarily collagen fibers (pink bundles), followed by a subcutaneous fat layer and striated muscle tissue (Figure 17(a)). To minimize bias, re‐epithelialization and granulation tissue formation were semi‐quantitatively scored on a 0–4 scale (0 = *absent*, 1 = *minimal*, 2 = *moderate*, 3 = *marked*, and 4 = *complete*) by two independent observers blinded to treatment allocation. Discrepancies were resolved by consensus.

Figure 17(a) Histological images of healthy skin at 40× and 400× magnifications, showing normal skin architecture: epidermis (E), hair follicle (F), dermal layer (L), muscle layer (M), and epidermal thickness (T). (b) Histological sections from Day 7 post‐wounding: Group I (negative control) displays severe inflammation and an open wound, while treatment groups, especially Group IV (PSH/ZnO NPs‐CH), show marked improvement. (c) Histological sections from Day 14: Group I still shows incomplete healing, whereas treatment groups, particularly Group IV, exhibit complete tissue regeneration, closely resembling healthy skin. Histological scoring of re‐epithelialization and granulation tissue formation was performed in a blinded manner.

**Figure:**
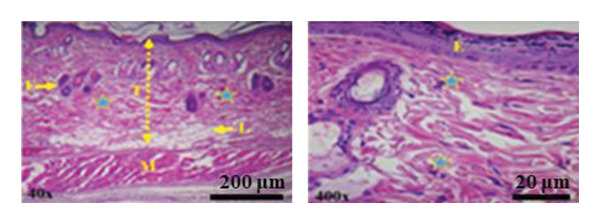
(a)

**Figure:**
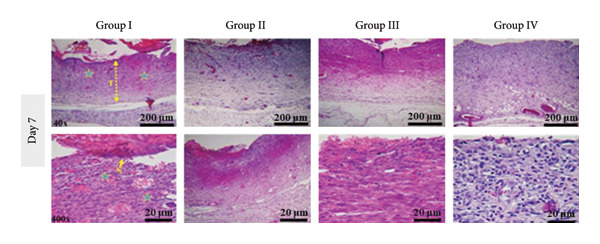
(b)

**Figure:**
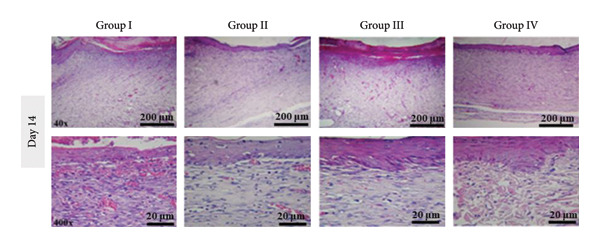
(c)

In Group I (negative control, treated with double‐distilled water), severe inflammation was evident on Day 7 (Figure 17(b)), marked by significant infiltration of immune cells, including neutrophils and macrophages. Angiogenesis was poor, and fibroblast proliferation and extracellular matrix (ECM) deposition were incomplete. By Day 14 (Figure 17(c)), slight improvement was noted, but the epithelial layer remained thin, and connective tissue regeneration was still deficient, indicating delayed healing. In Group II (positive control, treated with 1% phenytoin gel), inflammation was moderately reduced on Day 7 (Figure 17(b)), with increased fibroblast activity and granulation tissue formation. Enhanced angiogenesis suggested better healing progression compared to Group I, although the wound depth remained considerable, and epithelial regeneration was incomplete. By Day 14 (Figure 17(c)), this group showed superior regeneration, including a thickened epithelial layer, organized collagen deposition, and nearly complete tissue restoration. In Group III (treated with PSH hydrogel), a notable reduction in inflammation was observed on Day 7 (Figure 17(b)), along with early signs of epithelial regeneration. Fibroblast proliferation and collagen matrix formation were significantly improved, indicating a faster early‐stage healing response than phenytoin. However, by Day 14 (Figure 17(c)), although healing had progressed, the epithelial layer appeared less organized and structurally immature compared to the phenytoin‐treated group, and the collagen architecture remained poorly aligned. In Group IV (treated with PSH/ZnO NPs‐CH hydrogel), the most effective histological results were achieved. On Day 7 (Figure 17(b)), inflammation was substantially diminished, granulation tissue was rapidly forming, and angiogenesis was clearly evident. The presence of ZnO NPs likely promoted fibroblast proliferation and stimulated collagen synthesis. By Day 14 (Figure 17(c)), this group exhibited the most advanced tissue regeneration: A thick epithelial layer, well‐aligned collagen fibers, and intact blood vessels, signifying near‐complete wound closure and tissue recovery. These findings align with macroscopic healing observations. While PSH hydrogel outperformed phenytoin in reducing inflammation and initiating granulation tissue formation by Day 7, phenytoin demonstrated superior final‐stage regeneration by Day 14. Nevertheless, the PSH/ZnO NPs‐CH hydrogel formulation consistently yielded the most favorable healing response at both early and late stages, highlighting its strong potential as a superior wound‐healing therapeutic.

On Day 14, quantitative assessment revealed distinct differences in epidermal thickness across the groups. Normal skin displayed a mean thickness of 11.59 ± 1.89 μm. Group I showed the lowest value (6.17 ± 1.41 μm), reflecting delayed healing, whereas Group II exhibited partial improvement (9.81 ± 1.00 μm). Group III reached 14.63 ± 0.98 μm, suggesting excessive epidermal proliferation. The increased epidermal thickness observed in Group III likely reflects compensatory hyperplasia and enhanced keratinocyte proliferation induced by the bioactive components of PSH hydrogel, rather than fully organized tissue regeneration. Importantly, Group IV, which received the main therapeutic compound, demonstrated a thickness of 11.21 ± 1.70 μm, closely approximating the physiological baseline. Blinded scoring of histological sections further confirmed that Group IV exhibited the highest re‐epithelialization and granulation scores at both Day 7 and Day 14, consistent with quantitative thickness data. These findings indicate that, by Day 14, the treatment in Group IV promoted balanced and effective re‐epithelialization, restoring epidermal structure more appropriately than the other experimental groups.

## 4. Conclusion

In this study, we successfully developed a novel honey‐based PVA/SA hydrogel (PSH) incorporating green‐synthesized chitosan (CH)‐coated zinc oxide nanoparticles (ZnO NPs‐CH) aimed at enhancing wound healing. The integration of ZnO NPs‐CH into the PSH matrix produced a nanocomposite hydrogel (PSH/ZnO NPs‐CH) with improved physicochemical properties, including a more porous structure and controlled swelling, as confirmed by FE‐SEM, EDX, and ATR‐FTIR analyses. The hydrogel demonstrated significant antibacterial activity against both Gram‐negative *E. coli* and Gram‐positive *S. aureus*, indicating its potential to prevent wound infections. *In vivo* wound healing and histological studies showed that PSH/ZnO NPs‐CH notably accelerated wound closure, reduced inflammation, and enhanced tissue regeneration compared to controls. While the PSH hydrogel alone promoted early‐stage healing, the addition of ZnO NPs‐CH further improved both healing speed and final tissue recovery. These findings suggest that PSH/ZnO NPs‐CH hydrogel is a multifunctional wound dressing with excellent biocompatibility and antibacterial efficacy, offering substantial potential as an advanced wound care material. Nonetheless, further clinical trials and long‐term biocompatibility assessments are warranted to fully confirm its safety and effectiveness for clinical applications.

## Conflicts of Interest

The authors declare no conflicts of interest.

## Funding

No funding was received for this research.

## Data Availability

The data that support the findings of this study are available on request from the corresponding author. The data are not publicly available due to privacy or ethical restrictions.
